# Comparative Programs for Arthropod, Disease and Weed Management in New York Organic Apples

**DOI:** 10.3390/insects8030096

**Published:** 2017-09-04

**Authors:** Arthur Agnello, Kerik Cox, Jaume Lordan, Poliana Francescatto, Terence Robinson

**Affiliations:** 1 Department of Entomology, New York State Agricultural Experiment Station, Cornell University, Geneva, NY 14456, USA; 2 Plant Pathology and Plant-Microbe Biology Section, School of Integrative Plant Science, New York State Agricultural Experiment Station, Cornell University, Geneva, NY 14456, USA; kdc33@cornell.edu; 3 Horticulture Section, School of Integrative Plant Science, New York State Agricultural Experiment Station, Cornell University, Geneva, NY 14456, USA; jl3325@cornell.edu (J.L.); pf246@cornell.edu (P.F.); tlr1@cornell.edu (T.R.)

**Keywords:** high density, tall slender spindle, disease-resistant varieties, spinosad, azadirachtin, *Bacillus thuringiensis*, copper octanoate, *Bacillus amyloliquefaciens*, bark chip mulch, limonene

## Abstract

Organic apple production in the eastern US is small and is mostly based on existing varieties, which are susceptible to scab, and rootstocks, which are susceptible to fire blight. This requires numerous sprays per year of various pesticides to produce acceptable fruit. From 2014 to 2016, we tested different arthropod, disease and weed management programs in an advanced tall spindle high-density production system that included disease-resistant cultivars and rootstocks, in an organic research planting of apples in Geneva, New York. Arthropod and disease management regimens were characterized as Advanced Organic, Minimal Organic, or Untreated Control. Results varied by year and variety, but, in general, the Advanced program was more effective than the Minimal program in preventing damage from internal-feeding Lepidoptera, plum curculio, and obliquebanded leafroller, and less effective than the Minimal program against damage by foliar insects. Both organic programs provided comparable control of sooty blotch, cedar apple rust, and fire blight, with some variability across cultivars and years. The advanced selection CC1009 and Modi seemed to possess complete resistance to cedar apple rust, while Pristine had partial resistance. For weed control, bark chip mulch, organic soap sprays, and limonene sprays tended to be most effective, while mechanical tillage and flame weeding had lower success.

## 1. Introduction

Organic apple production continues to expand in the western US, but few growers have adopted the system in the eastern part of the country. In 2015, more than 4600 ha of apple orchards were reported to be under organic certification in Washington. However, in New York, the second largest apple producing state in the country, just over 50 ha were under organic management [1]. The reasons for the disparity in organic production between east and west are well documented [2,3,4] and include environmental (e.g., humidity and precipitation) and biological factors. In particular, eastern apple growers contend with more than 50 direct and indirect arthropod pests, as well as more than 20 plant diseases without the aid of effective pest management tactics.

Currently, the organic apple industry in New York is small compared with the state’s apple production, with an estimated 43 growers [1], compared with approximately 700 conventional commercial apple producers growing 16,200 ha of apples [5]. One of the main reasons there is so little organic apple production in New York (and other eastern states) is the difficulty of managing pests. The cost of arthropod and disease control under organic certification standards and its marginal effectiveness is a major obstruction to profitable organic apple production in New York State [2]. All commercial apple growers in New York must contend with diseases such as apple scab (*Venturia inaequalis* (Cooke) G. Winter) and fire blight (*Erwinia amylovora* (Burrill) Winslow et al.), and key insect pests such as oriental fruit moth (*Grapholita molesta* (Busck), codling moth (*Cydia pomonella* (L.)), obliquebanded leafroller (*Choristoneura rosaceana* (Harris)), plum curculio (*Conotrachelus nenuphar* (Herbst)), and apple maggot (*Rhagoletis pomonella* (Walsh)), plus numerous secondary fruit and foliar feeding insects and mites jointly capable of effecting as much as 100% crop loss if not effectively controlled.

Recent advances in integrated pest management, such as new formulations of organic copper and sulfur products, biopesticides, improved insect viruses, and pheromone disruption available for managing a larger number of insect pests and diseases, have not yet been comprehensively evaluated in organic apple systems, and could provide the missing components that have limited the success of organic apple production in the eastern US. Additionally, newly released apple scab-resistant cultivars have improved fruit quality, and could reduce the number of sprays required to manage apple scab. However, there are no commercially available cultivars bred to have resistance to blossom fire blight, powdery mildew, cedar apple rust, and summer diseases such as fly speck and sooty blotch. Hence, organic management options are needed for these diseases, which have become more endemic with apparent global climate change. Aside from diseases, there are also new options for weed management, as well as more precise methods for crop load management (thinning). Together, these new strategies need to be evaluated and incorporated into the high-density orchard production systems that have been proven to be highly profitable and labor-efficient under conventional management [6].

Previous work has established the relatively higher production costs of organic fruit systems compared with conventional systems [2,7]. When scab-susceptible cultivars are used in organic production systems, the use of sulfur, copper, and lime sulfur typically increases the total amount (by weight or amount) and cost of fungicides applied compared with conventional systems [7]. Moreover, even a recent study using disease-resistant trees [2] found that non-fungicidal (mainly insecticide) pesticide costs in an organic system were 30% higher than in the same trees managed under a conventional Integrated Fruit Production (IFP) system, which employed ecologically safer methods and pesticides than standard conventional systems. This was largely a result of the cost of kaolin clay; however, with a greater reliance on specialty products such as spinosad, pyrethrum and insect virus, the disparity in production costs was diminished. With a sustained market price premium for organic apples, some higher pest control costs could be acceptable. However, if the price premium should fall, less expensive control strategies would be essential.

New organic apple orchards, similar to conventional orchards, depend on rapidly filling the allotted space with canopy, and early fruiting, to pay back the initial investment of planting the orchard [8]. The best way to achieve high early yields is to plant high tree densities. Most new conventional orchards in NY are being planted in the tall spindle system, which has a suggested planting density of ~2965 trees/ha [9]. With high-quality nursery trees that have side branches and good tree growth in the first few years, significant yield can be achieved in the second year and full production by year five [6]. Such intensive orchards are not common among organic growers, which limits their profitability. In addition, almost all previous organic apple management research in the eastern US has been done with more traditionally spaced orchards [3,10]. Some organic growers lack an understanding of the economic benefits of high tree planting densities, while others believe larger, more vigorous trees are needed to overcome weed competition for water and nutrients. Other performance limitations in new organic orchards are the lack of good weed control, lack of intensive nutrient management, and lack of irrigation in the first two to three years.

Weeds can significantly affect tree performance, especially under organic management where inputs are often more limited. Therefore, if weeds are not effectively controlled, they can hinder tree growth and reduce yield, size, and fruit quality, which will affect the economic viability of the orchard in the long run [11,12,13,14]. Tillage, mulching, and mowing are the main strategies when it comes to organic weed management [12,15]. New organic herbicides may bring new opportunities, but results regarding the use of such herbicides have been quite inconsistent [15,16,17].

In this project, we used a newly planted high-density organic apple orchard of the most promising disease-resistant varieties to evaluate and demonstrate the most advanced and effective tactics available to the NY fruit industry for fruit production and organically acceptable management of arthropod and disease pests, as well to assess efficacy of mulching, mechanical tillage, flame weeding and organic herbicides to effectively control weeds under organic management.

## 2. Materials and Methods

### 2.1. Plot Layout

**Figure 1:**
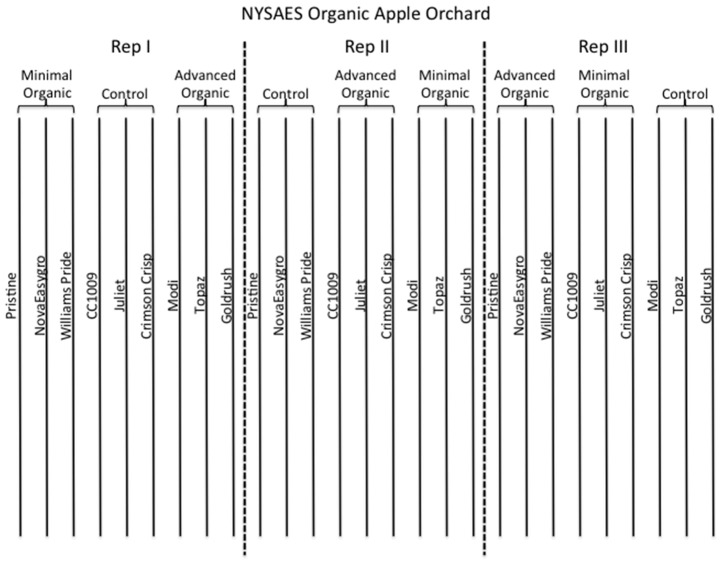
Layout of organic apple orchard showing varieties, treatments, and replicated plots, New York State Agricultural Experiment Station, Geneva.

Insect, disease, and weed management programs were compared over three growing seasons in an organic apple research orchard at Cornell’s New York State Agricultural Experiment Station in Geneva, New York. The site, composed primarily of Collamer silt loam, had been a fallow field with no previous history of fruit production, and there was no documented presence of any fruit diseases or insect pests before orchard establishment. The orchard was planted in the spring of 2012 and consists of a 1-ha (2400 trees) organic apple orchard containing replicated plots of nine disease-resistant varieties on two disease-resistant Geneva^®^ rootstocks: Pristine, Williams Pride, NovaEasygro, Crimson Crisp, Juliet, Modi, Topaz, Goldrush, and CC1009; the rootstocks used are G.202 and G.935. The plot was planted in a modern high-density tall spindle orchard system at a spacing of 0.9 × 3.7 m, giving a planting density of 2990 trees/ha (Figure 1). Trees have been trained as a slender fruiting wall. The plot has 3 replications with varieties in whole rows of 62 trees of each variety in each rep. No foliar nutrients or field-applied fertilizers were used during this study, and the site was not irrigated. Different arthropod and disease management regimens compared three treatment levels, set up in three replicates of 3-row plots (Figure 1: Layout of organic apple orchard showing varieties, treatments, and replicated plots, New York State Agricultural Experiment Station, Geneva.):

Advanced Organic: use of the most efficacious options available, although recognizing that these tactics and materials are likely to be more expensive and possibly more labor-intensive.

Minimal Organic: use of tactics that technically meet most common certification standards, but with a greater reliance on options that are more commercially available, less expensive and easier to implement, but possibly also less efficacious.

Untreated Control.

The Minimal organic program reflected the typical selection of products employed by local growers who endeavor to grow apples organically, being characterized by a general reliance on the most economical options available, and the Advanced program represented our attempt to find what we felt to be the most efficacious options possible, without regard for cost or even necessarily the practicality of implementing them (e.g., in regards to labor requirements, time needed to realize their full effects, etc.).

### 2.2. Arthropod Management

A season-long spray program was maintained each year, with applications made using a standard airblast sprayer calibrated to deliver an industry standard rate of 950 L/ha (100 gal/A), starting at bloom (2014 and 2016) or tight cluster (2015), depending upon spring weather patterns, and proceeding through August. The products applied in the Advanced Organic plots (Table 1: Organic Apple Spray Programs, 2014–2016) were: horticultural mineral oil (JMS Stylet-Oil, JMS Flower Farms, Vero Beach, FL, USA) for European red mite (ERM), *Panonychus ulmi* (Koch); *Bacillus thuringiensis kurstaki* (B.t.) (DiPel, Valent BioSciences, Libertyville, IL, USA) for obliquebanded leafroller (OBLR), *Choristoneura rosaceana* (Harris); spinosad (Entrust, Dow AgroSciences, Indianapolis, IN, USA) for codling moth (CM), *Cydia pomonella* (L.), OBLR, and apple maggot (AM), *Rhagoletis pomonella* (Walsh); kaolin clay (Surround, Engelhard, Iselin, NJ, USA) for plum curculio (PC), *Conotrachelus nenuphar* (Herbst); and 100% neem oil (Ahimsa Organics Neem Oil, The Ahimsa Alternative, Bloomington, MN, USA) for apple aphid, *Aphis pomi* De Geer, spirea aphid, *A. spiraecola* Patch, and potato leafhopper (PLH), *Empoasca fabae* (Harris).

The products applied in the Minimal Organic plots were: horticultural mineral oil for ERM; B.t. for OBLR and CM; pyrethrin (Pyganic, MGK, Minneapolis, MN, USA) for PC and AM; and azadirachtin (Aza-Direct, Gowan, Yuma, AZ, USA) for aphids and PLH.

Additional tactics were used in the Advanced Organic plots. On 8 July 2013, infective juveniles (IJs) of persistent northern-NY strains of *Steinernema carpocapsae* and *S. feltiae* entomopathogenic nematodes (EPNs) were applied to the soil surface using an ATV-mounted modified spray boom with five fertilizer (0010) nozzles and traveling at 5.6 km/h. In laboratory and field bioassays, these nematodes have shown efficacy as biological control agents against last-instar plum curculio larvae, which burrow in the soil to pupate [18]. Because these strains are native to New York State, they are adapted to persist and eventually spread to untreated soil under our typical climatic conditions. The application of a 50:50 combination of *S. carpocapsae* and *S. feltiae* IJs was made to the orchard rows and row middles of the three 3-row Advanced Organic plots, at a rate of approximately 11 billion total IJs/ha. Soil core samples taken from the treated rows annually since then and assessed for EPNs provided evidence of nematode establishment by the end of the 2015 season.

In addition, during the 2012 and 2013 growing seasons, predator mites (*Typhlodromus pyri* (Scheuten)) were collected from foliar terminals in a nearby research orchard and introduced over a period of several weeks on leaf discs (10 per disc) affixed to shoots in the Advanced Organic plots. The predator mites were introduced into trees of the middle rows in each target plot on six dates in July–August 2012 and on five dates in June–August 2013.

Pheromone traps were deployed to track flights of the major Lepidoptera pest species (oriental fruit moth, codling moth, obliquebanded leafroller), and fruit and foliar samples were taken at regular timings to evaluate insect pest presence and damage in the different treatments. For PLH and the green aphid complex (apple aphid and spirea aphid), three foliar terminals on each of 10 trees in the middle rows of each treatment plot were examined for the presence of infestations, and the results expressed as percent infested terminals.

Samples were taken to assess infestations of overwintered and summer broods of OBLR, as well as internal-feeding Lepidoptera such as codling moth, oriental fruit moth and lesser appleworm (LAW), *Grapholita prunivora* (Walsh). For the 2014 and 2015 overwintered OBLR samples, 10 trees per row were inspected for the presence of larvae during the late bloom-fruit set period, and the percentage of trees showing infestations was recorded. In 2016, because the trees were older and more established, 10 foliar terminals on each of 10 trees per row were examined to determine the mean percent damaged terminals in each treatment. In 2014 and 2015, we examined 10 fruits per tree on each of 10 trees per row, and the percentage of fruits with feeding damage caused by either OBLR or internal-feeding Lepidoptera was recorded.

Foliar samples were taken once each year during early August in 2015 and 2016 to assess populations of both predacious (*T. pyri*) and phytophagous mites (ERM and twospotted spider mite, *Tetranychus urticae* Koch). On each sample date, 25 intermediate-age leaves were picked at random from shoots on five trees (5 per tree) per row, and brushed in a mite-brushing machine to determine the numbers of phytophagous mite eggs and mite motile forms plus predacious mite motiles in each 25-leaf sample.

Insect damage at harvest was assessed each year by inspecting 100 fruits randomly picked from trees in each row of each plot (i.e., one row per treatment per variety) to determine mean percent fruit damage by treatment across varieties caused by each direct fruit pest. All varieties were sampled in 2014 and 2015, but, due to late spring frost damage and drought conditions during the 2016 growing season, only five varieties had sufficient fruit to include in the analysis that year.

### 2.3. Disease Management

Season-long trials were conducted to evaluate the effectiveness of organic fungicides and bactericides against the sooty blotch/flyspeck (2014, 2015, and 2016) late season summer diseases, cedar apple rust (2015 and 2016), a mid-season disease, and fire blight, a bacterial disease that causes infection during bloom (2015 and 2016) (Table 1: Organic Apple Spray Programs, 2014–2016). Disease management programs began at tight cluster (2015 and 2016) or 2nd cover (2014), depending on spring weather, and continued through harvest in August. Applications of fungicides for flyspeck/sooty blotch and cedar apple rust were made using an airblast sprayer calibrated to deliver an industry standard rate of 950 L/ha (100 gal/A). Treatments for fire blight were applied using a gas-powered backpack sprayer (Solo 451, Solo Inc., Newport News, VA, USA). These sprayers were calibrated to deliver dilute (2850 L/ha [300 gal/A]) applications to runoff using the high droplet setting to facilitate the high-volume application. Fire blight applications were made at 80% bloom and full bloom to the Topaz, Modi, and Goldrush blocks. These cultivars were chosen for their susceptibility to fire blight and to minimize the extent of planting-wide impact. Trees were inoculated at full bloom with *Erwinia amylovora* strain Ea 273 at 1 × 10^4^ CFU mL^−1^ using a hand pump backpack sprayer (Solo, Inc.) [19,20].

The products applied in the Advanced Organic plots (Table 1) were: Copper Octanoate (Cueva 7 L/ha or 4.67 L/ha, Certis USA, LLC, Columbia, MD, USA), *Bacillus amyloliquefaciens* d747 (Double Nickel LC 2.3 L/ha, Certis USA) and the products applied in the Minimal Organic plots were: copper hydroxide + copper oxychloride (Badge X2 5.6 kg/ha, Gowan Company, Yuma, AZ, USA), and sulfur (Microthiol Disperss 16.8 kg/ha, United Phosphorus, Inc., King of Prussia, PA, USA). All treatments were made to three replicate blocks containing a minimum of 15 trees per block. Please confirm if it is right.

The incidence of blossom blight was evaluated on 3 June in 2015 and 1 June in 2016. The incidence of blossom blight was expressed as the number of blighted blossom clusters out of 5 clusters, with 20 collections of clusters assessed for each treatment replicate. The incidence of sooty blotch/flyspeck symptoms on mature fruit was assessed at harvest. The incidence of cedar apple rust symptoms on terminal leaves was assessed on 19 August in 2015 and 23 August in 2016, and the incidence of cedar apple rust symptoms on mature fruit was assessed at harvest. The incidence of sooty blotch and cedar apple rust on mature fruit was calculated as the number of mature fruit with flyspeck or sooty blotch, out of five sampled fruits, with 10 such samples assessed for each treatment replicate. The incidence of cedar apple rust symptoms on terminal leaves was calculated from the number of terminal leaves with cedar apple rust lesions with pycnidia out of eight fully expanded leaves from the distal end of the shoots, with 10 shoots assessed for each treatment replicate [21].

### 2.4. Weed Management

The weed management trial was designed as a separate experiment overlaid in a balanced design on each rep of the insect and disease treatments, with a strip split-plot design. Each row in each insect and disease treatment was subdivided into 7 subplots of 7 trees each with subplot weed control treatments laid out in strips across all 9 rows of the insect and disease rep. The weed control experiment was designed as a randomized complete block experiment with 3 replications of 9 rows each with plots of 7 trees in each row. Thus, the weed control experiment was balanced across each insect/disease plot and did not confound the results or analysis of the insect/disease treatments. Weed control treatments compared seven treatments applied to each of the nine scab-resistant varieties and were performed during 2014–2016. Weed control treatments were: (1) Flame weeding along each side of the tree using a propane flame machine; (2) Organic limonene (Avenger, Avenger Organics, Buford, GA, USA); (3) Mechanical cultivation on each side of the tree using a cultivator (Wonder Weeder®, Burbank, WA, USA); (4) Organic soap (Final-San-O, Columbia, MD, USA); (5) Organic acetic acid (Weed Pharm, Port Townsend, WA, USA) (2014 only); (6) Bark chip mulch 20-cm deep along the tree row; and (7) Caprylic acid (Suppress, Westbridge, Vista, CA, USA) (2015–2016 only). Each of the treatments was applied every 3 weeks during the season starting in late May (total of 5 applications). For the organic herbicides, the rate applied was as recommended on the label. Weed control was assessed by determining percent weed-free area in the 2-m-wide and 7-m-long weed control strip under the trees at two times during the season in 2014–2015 (11 August and 1 October), and once in 2016 (1 September). After the first weed control assessment in August (2014–2015), all the plots were hand-weeded, then a second round of treatments was applied in August and September and plots were re-assessed on 1 October.

### 2.5. Data Analysis

The trials assessing efficacy of the treatments in each discipline (arthropods, diseases, and weeds) were analyzed independently of each other, and each year was analyzed individually. For the insect data, a one-way ANOVA was used with treatment as the main factor with three replicates as random factors. Mean treatment effects were separated using Student’s *t*-test, using the JMP statistical software package (Version 12; SAS Institute Inc., Cary, NC, USA). Disease incidence data was analyzed separately for year and cultivar and was subjected to analysis of variance (ANOVA) using Generalized Linear Mixed Models with the GLIMMIX procedure of SAS (version 9.4; SAS Institute Inc.) with treatments as fixed effects and the replicate blocks as random effects. All percentage data were subjected to arcsine square root transformation prior to analysis to stabilize the variances. For the weed treatments, weed-free area was modeled using linear mixed effect models. Mixed models including treatment as a fixed factor, and block and variety as random factors were employed to separate treatment effects. For all the models, when the main effect (treatment) was significant, comparisons among treatments were made by Tukey’s HSD test at *p* values ≤0.05 using the JMP statistical software package or the LSMEANS procedure in SAS (version 9.4).

## 3. Results

### 3.1. Foliar Arthropods

The in-season sampling sessions for infestations of overwintered obliquebanded leafroller larvae showed no difference among treatments in 2014 or 2015, but, in 2016, trees in the Advanced Organic program had significantly lower infestations than the Untreated Control, while those in the Minimal Organic treatment were statistically comparable to both the Advanced and Control treatments (Table 2: Obliquebanded leafroller and fruit-feeding Lepidoptera in-season damage). A similar trend was seen among treatments for fruits damaged by internal-feeding Lepidoptera (codling moth, oriental fruit moth and lesser appleworm) in 2014 and 2015; damage levels in the Minimal Organic program fell between, but did not differ from, the other treatments.

Conversely, the Advanced Organic performed no better than the Minimal Organic treatment in preventing terminal infestations of green aphids during all three years, and neither differed significantly from the Untreated Control (Table 3: Green aphid and potato leafhopper terminal infestations under different organic programs). In 2014, the 23 June spray of Neem oil and Aza-Direct had some impact on the pre-treatment aphid populations in the Advanced Organic and Minimal Organic plots, respectively, but infestation levels were not statistically lower than those in the Untreated Control plots, either immediately post-treatment or throughout the duration of the season (Table 3). The same trend was observed in 2016 after the 30 June spray applications in these plots. Foliar infestation levels were uniformly low in 2015, irrespective of the management program.

Foliar populations of potato leafhopper in 2016 were reduced to zero by the spray application on 30 June, which was statistically lower than the levels remaining on the Untreated Control trees (Table 3).

**Figure 2:**
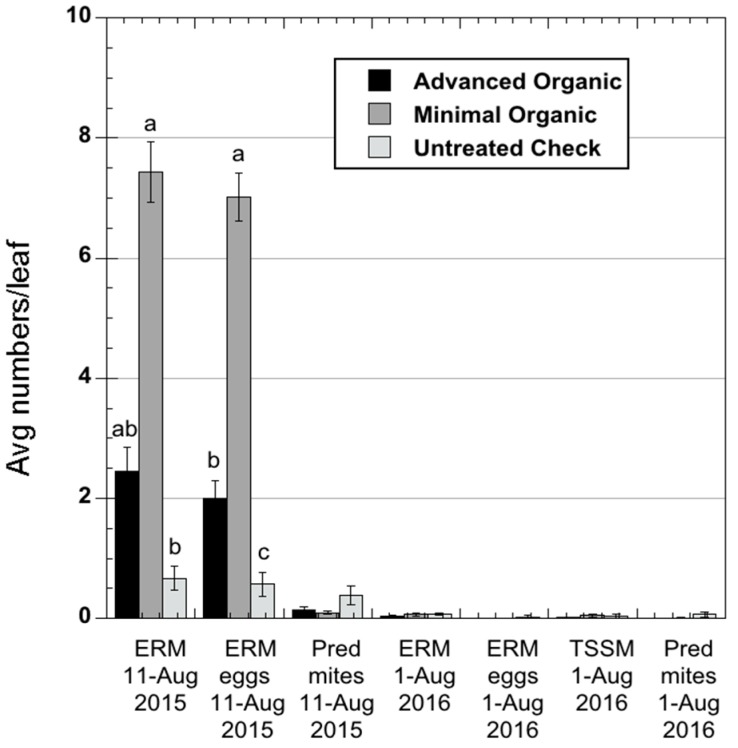
Average numbers per leaf of eggs and motile forms of European red mite, *Panonychus ulmi* (Koch), the predacious mite, *Typhlodromus pyri* (Scheuten), and twospotted spider mite, *Tetranychus urticae* Koch, in once-yearly foliar samples taken during 2015 and 2016.

Mite populations were higher in 2015 than 2016, but never reached economically damaging threshold levels either year (August threshold = 7.5 mites/leaf [22]). The foliar mite sample on 11 August 2015 (Figure 2: Average numbers per leaf of eggs and motile forms of European red mite, *Panonychus ulmi* (Koch), the predacious mite, *Typhlodromus pyri* (Scheuten), and twospotted spider mite, *Tetranychus urticae* Koch, in once-yearly foliar samples taken during 2015 and 2016) showed below-threshold European red mite populations in both organic treatment regimens by the end of the season, but the numbers of motile forms and eggs in the Advanced Organic plots, as well as in the Untreated Control, were lower. Predator mite levels were similarly low, and comparable in both treatments, although numerically somewhat higher in the Untreated Control. In 2016, there were low populations of all mite species and life stages throughout the summer, with no significant differences seen among treatments.

#### Fruit Harvest Evaluations

In terms of overall fruit insect damage present at harvest throughout this study, the mean damage ratings were somewhat variable according to variety, but the highest pooled levels of damage were seen in the categories of plum curculio oviposition and feeding, and internal Lepidoptera (mainly codling moth) infestation and obliquebanded leafroller feeding (Table 4). In 2014, no significant differences were seen among treatments in damage caused by PC oviposition or feeding, or by internal-feeding Lepidoptera; only late-season OBLR damage was statistically lower in the Advanced Organic plots than in the Untreated Control, with damage in the Minimal Organic plots again comparable to both.

The Advanced Organic program had significantly lower PC oviposition damage than the Untreated Control in 2015 and 2016, and also lower PC feeding damage in 2016; internal Lepidoptera damage was also significantly higher than in the Minimal Organic treatment in 2015, but rosy apple aphid and was higher, as was San Jose scale fruit damage in 2016 (Table 4: Percent fruit insect damage at harvest under different organic programs, 2014–2016). Overall percent clean fruit in the Advanced Organic plots ranged 50.5–62.6% throughout the study, which was significantly higher than in the Untreated Control in 2015–2016 (range 16.6–42.4%), and also higher than in the Minimal Organic plots (at 44.3%) in 2015 (Table 4).

Although no statistics were run on the results among different varieties because of insufficient replication, the Advanced Organic plots had generally higher overall numerical levels of clean fruit in the varieties Crimson Crisp, Modi, Topaz, and Goldrush; however, the Minimal Organic clean fruit levels were higher in Pristine, Nova Easygro and Juliet. The Advanced Organic program was generally more effective than the Minimal Organic program in the category of internal Lepidoptera/OBLR damage, and less effective than the Minimal Organic program against plum curculio oviposition [23].

### 3.2. Diseases

Across all three years and cultivars, the incidence of sooty blotch/fly speck ranged 0–100%, with the higher incidences observed in 2015 compared with 2016 (Table 5: Development of sooty blotch and flyspeck in selected varieties under different organic management programs, 2014–2016). In all years, the highest incidences of sooty blotch/fly speck were observed on the cultivars Goldrush and Topaz, while the lowest incidences were observed on Juliet, Modi, and Crimson Crisp. Across all cultivars and years, both organic programs had statistically lower incidences of sooty blotch/fly speck compared with the Untreated Control (*p* < 0.05). The two organic programs provided a statistically similar level (*p* > 0.05) of fly speck/sooty blotch control except on Goldrush in 2014, where the Advanced Organic program was more effective, and Topaz in 2015, where the Minimal Organic program performed better.

Of the two years that cedar apple rust developed, its incidence on terminal leaves ranged 0–77%, with higher incidences of rust observed in 2015 compared with 2016 (Table 6: Development of cedar apple rust on terminal leaves in selected varieties under different organic management programs, 2015–2016). In all years, the highest incidences of cedar apple rust were observed on Goldrush, Crimson Crisp, and Topaz, while the lowest incidences were observed on Juliet, Pristine, Modi, and CC1009. Interestingly, CC1009 and Modi seemed to be completely resistant to cedar apple rust, despite the proximity to cultivars with high incidences of rust. Both organic programs had statistically lower incidences of cedar apple rust compared with the untreated check (*p* < 0.05). The two organic programs provided a statistically similar level of cedar apple rust control on all cultivars in both years (*p* > 0.05) and the level of control was such that premature defoliation was not observed. In both years, cedar apple rust lesions were also observed on fruit. The incidence of cedar apple rust on fruit was typically lower than on leaves, but the trends for rust development on fruit were identical to those on leaves [24].

In 2015, the incidence of blossom blight ranged 0–7% and the incidence of blossom blight observed in 2016 ranged 0–24% (Table 7: Development of blossom blight in selected varieties under different organic management programs, 2015–2016). Unlike the two fungal diseases, there were no clear differences in the susceptibility of the three cultivars to fire blight (*p* > 0.05). Both the Minimal and Advanced Organic programs had statistically lower incidences of blossom blight compared with the Untreated Control (*p* < 0.05) and provided a statistically similar level of control on all cultivars in both years (*p* < 0.05).

### 3.3. Weeds

**Figure 3:**
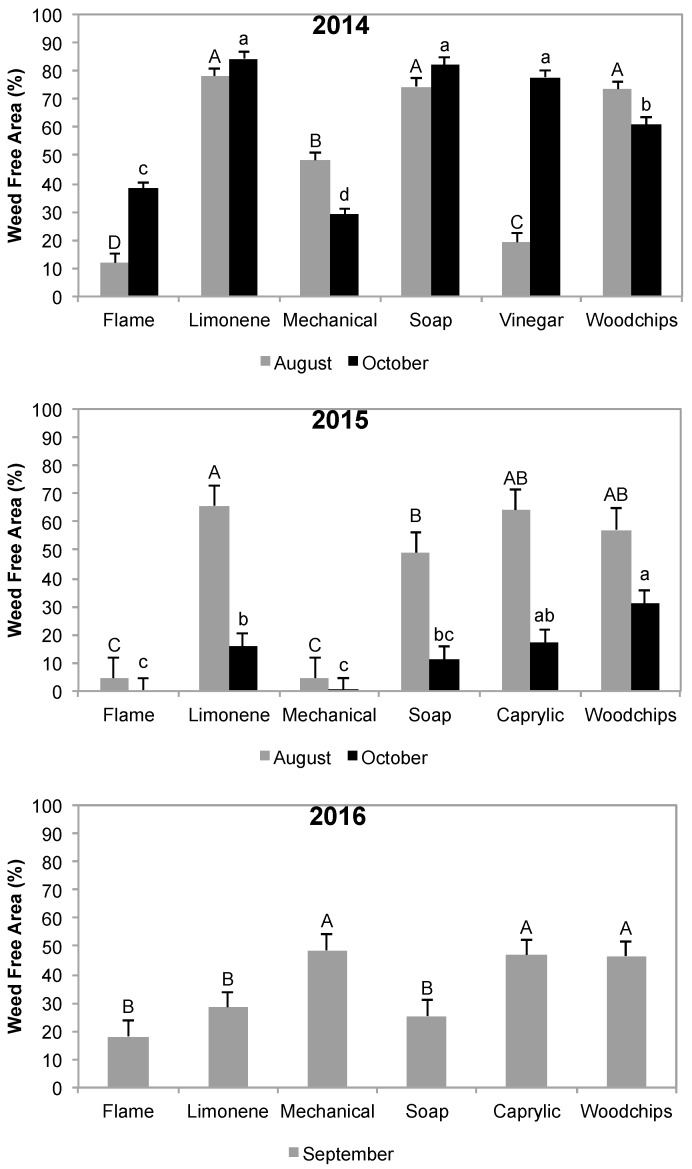
Effect of various organic weed control methods on the production of weed-free area (%) along the tree row during 2014–2016. Bars with different letters denote significant differences among treatments for each period (August, October, or September) (Tukey’s honestly significant difference, *p* ≤ 0.05).

In 2014, the limonene and soap sprays, and the wood chips, gave statistically the best early season weed control (Figure 3: Effect of various organic weed control methods on the production of weed-free area (%) along the tree row during 2014–2016). However, in the part of the field where Canadian thistle (*Cirsium arvense* (L.) Scoop, Asteraceae) was a problem, even the wood chip mulch was not enough to prevent growth of this weed. The monthly sprays of an organic soap and the organic limonene product gave excellent weed control (~80% weed-free area). Monthly mechanical tillage or monthly flaming provided statistically lower weed control (40–50%) that left a 25 cm-wide strip along the row, which caused significantly poorer tree growth. Monthly sprays of an organic vinegar product gave statistically poorer weed control efficacy in the early season (20%), but significantly better weed control in the late season (78%).

In 2015, mechanical and flame weed control strategies did not control weeds along the tree row (Figure 3). The wood chips treatment gave statistically better weed control but maintained an excessively high level of soil moisture, which hampered tree yield. In addition, the limonene and caprylic acid treatments allowed more than half of the area to remain free of weeds, while, for the soap, it was around 50%.

In 2016, the mechanical, caprylic acid, and wood chips treatments gave statistically the best weed control, with about 50% of the area remaining weed-free (Figure 3). Similar to previous years, flaming gave the lowest control, whereas soap and limonene were not as efficient as reported in the first two years of testing.

## 4. Discussion

### 4.1. Arthropods

Previous studies on organic apple production in the eastern US have demonstrated the potential for year to year variability in arthropod damage to both the fruit and foliage, depending on seasonal rainfall and temperature patterns and their effects on local insect and mite populations. Although plum curculio and internal-feeding Lepidoptera uniformly caused the greatest amounts of fruit damage in all three years of this study, most other insects had quite variable impacts on fruit quality, depending on the year. For instance, tarnished plant bug and rosy apple aphid damage was higher in 2015 than in the other years, obliquebanded leafroller late-season damage was worst in 2014, and stink bug damage did not occur at any measurable level until 2016 (Table 4). Peck et al. [2], who implemented a similar insect management program, had comparable variability over a three-year period with regard to internal Lepidoptera and tarnished plant bug damage. Similarly, Berkett et al. [4] found distinctly different levels of damage in different years from most pests, including tarnished plant bug, leafrollers, plum curculio and internal-feeding Lepidoptera. Peck et al. [2] noted the importance of continually having to adapt and modify their insect pest control strategies. Our overall levels of clean fruit during this study (50.5–62.6% in the Advanced plots) were comparable to those found by Berkett et al. [4] over three years (35.3–68.3%), and more uniform than those seen by Peck et al. [2] over four years (25–97%).

Incidence of foliar-feeding arthropods in our study appeared not to be influenced as significantly by the management program employed (Table 3). Aphid infestations tended to be minor across treatments all three seasons, and European red mite populations were not observed to exceed economic damage thresholds as commonly as in conventionally managed orchards (Figure 2). Predacious mites were present at low levels across all the treatments, even in the plots that were not seeded with motiles, perhaps indicating that the effort to seed them was not necessary. The general low-level of toxicity to non-target species of most of the insecticides used during this study may have contributed to this outcome, as this would be expected to result in less disruption of the natural enemy populations, although some products used, such as kaolin, sulfur, and spinosad, may have had some detrimental effects on predatory mites, which could have interfered with population establishment. Potato leafhopper, which migrates into the region each year on weather fronts, differed in this regard, as infestations during 2015 and 2016 were severe enough to potentially cause significant leaf damage (Table 3).

Because this orchard has been under organic management practices for only a few years, it is certain that we have not yet encountered all of the possible challenges inherent in organic apple production in New York. Insect populations can be expected to continue to develop, and new species and infestation patterns will likely necessitate additional measures, such as pheromone mating disruption or insect virus for internal fruit-feeding Lepidoptera, or specialized trunk treatments for trunk-boring beetles. Because of this orchard’s young age, no apple maggots were detected until the third season, and only at low levels, in just the Untreated Control plots; however, this is expected to be a more serious and predictable pest as the trees mature and become capable of more typical levels of fruit production. Biopesticides may offer some useful new alternatives for arthropod management in organic apple production in future years, even in regions with high pest population pressure such as New York, and the eastern US in general. In this study, we relied on the most well-known representatives of this class of insecticides: *Bacillus thuringiensis*, azadirachtin, and pyrethrin. However, others have been registered and are being evaluated for efficacy in various crop systems, and some of them may ultimately prove effective against selected orchards pests typically found in this region. Along with more familiar active ingredients such as *Beauveria bassiana* and *Metarhizium brunneum* [25], there are several new products being evaluated that contain extracts of, e.g., *Chromobacterium subtsugae* and *Burkholderia* spp. [26,27,28]. More products are under development, but have yet to be thoroughly evaluated in apple systems.

### 4.2. Diseases

There have been a few studies evaluating disease management in organic plantings of apples [2,4]. While these studies were principally systems evaluations of organic fruit production in the eastern US, they did involve comprehensive disease management evaluations. All of the cultivars in the present study had the *Rvi7* gene from *Malus × floribunda* 821 for resistance to apple scab [29]. In North America, there are no reports of stable populations of *Venturia inaequalis,* the causal agent of apple scab, with the avirulence gene *AvrRvi7*. Apple scab was never observed on resistant cultivars during the trials years, but considerable development of mature apple scab lesions, beyond the hypersensitive response expected for *Rvi7* cultivars, was observed in the 2017 season following long extended periods of rain with seasonal rainfall exceeding 30 cm. We were unable to determine if this was due to a breakdown of resistance or possibly from overwhelming the gene-for-gene interaction of *Rvi7* and *AvrRvi7* with inoculum and disease pressure. Despite resistance to apple scab in the cultivars, the presence of other early season fungal diseases such as cedar apple rust and summer diseases such as sooty blotch and flyspeck warranted the use of a full season fungal disease management program.

In the present study, sooty blotch/fly speck was found to be an endemic problem and, when left untreated, rendered nearly 70–80% of the fruit unmarketable. The studies by Berkett et al. [4] and Peck et al. [2] also reported development of fly speck and sooty blotch at incidences ranging from 0% to 10% in organic plantings, which were managed with applications of lime sulfur. Although we evaluated different varieties, we did achieve similar levels of control with sulfur in the Minimal Organic program and a low rate of a copper soap plus a *Bacillus*-based biological control agent in the Advanced Organic program. Moreover, the presence of untreated trees in the current study indicates that we were able to achieve such control in the presence of high disease pressure (>70% incidence) that would have nearly ruined the marketability of the crop in the absence of a management program.

Cedar apple rust can be devastating in orchards when conventional fungicides are absent or when orchards are planted near juniper trees. Peck et al. [2] did not observe any cedar apple rust in their study, but Berkett et al. [4] observed high incidences of cedar apple rust (>30%) on foliage and fruit in all three years of their study, despite applications of lime sulfur for disease management. In the present study, we were able to achieve high levels of cedar apple rust control in nearly all cultivars under both the Minimal and Advanced Organic programs, despite the high cedar apple rust pressure in the Untreated Control. Similar to Berkett et al. [4], we also found variability in susceptibility to cedar apple rust. We found that Modi and CC1009 were completely resistant, and that Prisitine and Juliet were less susceptible and had cedar apple rust incidences similar to that reported for Liberty, Macoun, and Zestar! by Berkett et al. [4]. The cultivars Crimson Crisp, Goldrush, and Topaz were highly susceptible and had incidences of cedar apple rust similar to Ginger Gold and Honeycrisp, as reported by by Berkett et al. [4]. Further work is needed to identify the cultivars with natural resistance to cedar apple rust to strategically deploy cultivars in organic plantings in the northeastern US where juniper trees are endemic.

Fire blight developed following flower inoculations in 2015 and 2016. The incidences of blossom blight were typically lower than those in other published trials [19,20]. This was likely due to the lower dose of 1 × 10^4^ CFU·mL^−1^ used in these trials compared with a standard of 1 × 10^6^ CFU·mL^−1^ [19,20]. A lower dose was chosen for these trials to better mimic a natural level of inoculum experienced by growers in a high-pressure year, instead of simply evaluating relative product performance, which requires high inoculum levels to separate fine differences in performance. As would be expected from previous studies [19,20,30], organically approved coppers and biologicals were unable to provide the high levels of control typically achieved with conventional antibiotics that are no longer allowed for organic apple production. However, this study and others [19,20,30] have shown that organically approved options can still provide a substantial level of blossom blight control when timed at the periods of high infection risk. Given the levels of blossom blight observed in our study, it would be essential for growers to scout and remove any fire blight to ensure the sustainability of the planting, despite implementation of a chemical or biological management program for fire blight.

Together, the current study and the previously published studies begin to establish a base of information for disease management in organic fruit production independent of the level of apple scab and general disease resistance in cultivars marketed for organic production. Moreover, new biopesticides are being developed or optimized for improved disease control. Such materials include natural systemic acquired resistance (SAR) inducers such as *Reynoutria sachalinensis* or *Bacillus mycoides*, anti-microbial metabolites produced from *Bacillus subtilis* and *Bacillus amyloliquefaciens*, and those produced by Lupins (Banda de *Lupinus albus* doce; BLAD). Such formulated products have been found to provide promising levels of control of blossom and shoot blight of fire blight [19,20,31]. Along these lines, there are many more naturally derived copper products being developed that optimize the effective dose of copper such as the ones included in the is study (copper octanoate and copper hydroxide + copper oxychloride) and copper sulfate pentahydrate, which has been shown to work well with bio pesticides with noticeable injury when applied at bloom or shortly after [20,31]. All that said, little is known on the effectiveness of the aforementioned biopesticdes on the fungal diseases investigated in this study. More work is needed to understand their full potential and optimal use practices for other diseases of apple.

### 4.3. Weeds

Overall, flame burning provided the poorest weed control. Based on our observations, flaming might work better in drier climates (average rainfall in Geneva, NY, USA from June through September is 343 mm) where irrigation stress is most likely, thus weed regrowth after flaming would be hampered. Similar to our results, flaming was reported not to be very efficient in other studies, especially in controlling larger rhizomatous weeds [13,15]. However, flaming was observed to be very effective by Stefanelli et al. [32], although these authors also suggested that it may damage branches and irrigation lines if not applied properly.

Mechanical control was also not very efficient in our study, as our abundant and regular rainfall requires mechanical weeding to be more frequent. In addition, some authors have also reported that growth of certain weeds can even be stimulated by tillage [33]. Nevertheless, mechanical tillage was observed to provide slightly better weed control than organic herbicides, as reported in other studies [16]. In our trial, wood chips worked better than mechanical tillage in terms of weed-free area. The effectiveness of wood chips in controlling weeds has been widely reported [12,34,35], along with their effect on promoting tree growth [34]. However, others [11] reported poor control of perennial weeds. In our case, weed control was not satisfactory when Canadian thistle was present, and also poor tree growth was observed, due to excessively high moisture. This effect might not be a problem in drier areas such as the Pacific Northwest [34,36], but in cooler, humid climates such as New York, it could become significant.

Overall, the best weed control in our study was provided using limonene, soap, and caprylic acid sprays, whereas vinegar was not consistent throughout the season. Inconsistency of vinegar has been previously reported in other studies [37]; however, these authors suggested that inconsistency of vinegar in controlling weeds could be improved by applying it at high temperatures and relative humidity. Variable results were also observed by other researchers for caprylic acid [38], where the best weed control included a combination of organic herbicide plus mowing. Limonene and soap were not as effective in 2016 compared with 2014–2015. In previous years more sprays were applied, which suggests that these two treatments work well, but need more applications to be comparable to caprylic acid. Therefore, more frequent sprays might have increased the effectiveness of the limonene, soap, and even caprylic acid treatments. Limonene has been reported to provide very efficient weed control in almond orchards in California [17], whereas poor results were observed in vineyards, with acceptable control lasting only 2–3 weeks [16]. The need for more frequent treatments for effective control of organic herbicides has likewise been suggested [17,37]. Efficacy of organic herbicides will strongly depend on region, climate, and the type of weed [15]. Thus, the need to perform local experiments to see what works best for each situation becomes crucial for effective weed control. In conclusion, frequent sprays (≥5 per season) of limonene or caprylic acid would be the most suitable strategy for organic apple production in cool, humid climates such as New York.

### 4.4. Program Costs

Records were kept of the per-hectare costs associated with each of the arthropod and disease spray materials applied each year (Table 8: Costs of arthropod and disease control products sprayed under different organic management programs, 2014–2016). In 2014, the cost of the Advanced Organic insect management program (US$1166/ha) was only 7.7% higher than that of the Minimal Organic program (US$1088/ha). The Advanced Organic disease management program (US$558/ha) was nearly 49% more expensive than the Minimal Organic program (US$375/ha). The overall cost of these combined Advanced programs (US$1724/ha) was 18% higher than the combined cost of the Minimal programs (US$1457/ha). In 2015 and 2016, the cost of the Advanced Organic insect management program (US$1344/ha) was 14% higher than that of the Minimal Organic program (US$1176/ha). The Advanced Organic disease management program (US$1032/ha) was twice as expensive as the Minimal Organic program (US$514/ha). The overall cost of these combined Advanced programs (US$2376/ha) was 29% higher than the combined cost of the Minimal programs (US$1690/ha). These costs are actually fairly comparable to those incurred in orchards under conventional production; a 2014 Cornell Farm Business Survey conducted by the Cornell Cooperative Extension Lake Ontario Fruit Program [39] gives the average pesticide cost (including insecticides, fungicides, plant growth regulators, foliar nutrients and adjuvants) as US$1959/ha (range, US$1319–$3159/ha). However, depending on the intended market for the fruit, there was likely still more than an acceptable level of fruit damage in most of the varieties, especially in consideration of the associated control costs and increased labor, especially in the advanced program.

## 5. Conclusions

This study was an attempt to evaluate and compare specific selected combinations of organically acceptable tactics and products in their efficacy against the complex of arthropods, diseases and weeds occurring in a typical New York apple production system. Designation of the individual elements in each management program as either Minimal or Advanced was admittedly somewhat subjective, and was based on our respective familiarity with measures commonly employed or proposed as options against each category of pests for organic apple production, but treatment levels were intended to represent the most realistic partitioning of economic and efficacy criteria likely to be used under typical production scenarios. This necessarily limited the scale of our comparisons, in both spatial and temporal terms, and likewise made it impractical to adequately examine interaction effects both within and across pest categories that could confound efforts to make more reliable long-term conclusions. Nevertheless, the levels of efficacy we found against pest species in the respective categories (arthropods, diseases, and weeds) were variable enough, and in some cases equivocal enough, regardless of treatment, to corroborate the prevailing view that the economics of organic production will be dictated by the existence of a suitable market for the crop. A packout of 50–60% clean fruit, while patently unacceptable in conventional production, would easily justify the increased costs of some organic programs if the economic returns were sufficient. The role of researchers in areas of crop protection is to evaluate the most promising and practical options available, and propose methodologies to optimize their success.

## Figures and Tables

**Table 1:** Organic Apple Spray Programs, 2014–2016.

Target	Timing	2014	2015	2016	Product and rate/ha	
					Advanced Organic	Minimal Organic
*Arthropods*						
Mites	Tight Cluster	-	30 April	-	Stylet Oil (18.7 L)	Stylet Oil (18.7 L)
OBLR	Bloom	22 May	11 May	17 May	Dipel (1.12 kg)	Dipel (1.12 kg)
Codling moth, OBLR	PF + Covers	29 May, 14 June	18 May, 25 June, 20 July	24 May, 7 and 30 June, 29 July, 9 August	Entrust (438 mL)	Dipel (1.12 kg)
Plum Curculio	PF + Covers	29 May, 10 June	18 and 26 May, 4 June	24 May, 7 June	Surround (56 kg)	Pyganic (4.67 L)
Aphids, PLH	mid-season	23 June	6 July	30 June	Neem oil ^a^ (9.35 L)	Aza-Direct (1.46 L)
Apple maggot	late season	1 and 15 August	4 August	29 July, 9 August	Entrust (438 mL)	Pyganic (4.67 L)
*Diseases*						
Inoculum of fungal and bacterial	Green Tip	25 April	-	-	Microthiol Disperss (16.8 kg)	Microthiol Disperss (16.8 kg)
diseases			22 April	17 April	Cueva (7.0 L)	Badge X2 (5.6 kg)
Powdery mildew and Fire blight	Pink	-	8 May	6 May	Cueva (7.0 L)	Badge X2 (1.4 kg)
Powdery mildew and Fire blight	80% Bloom	-	12 May	11 May	Cueva (4.67 L) + Double Nickel (2.34 L)	Badge X2 (1.4 kg)
Sooty Blotch/Flyspeck and Fire blight	Late Bloom to Petal Fall	-	15 May	23 May	Cueva (4.67 L) + Double Nickel (2.34 L)	Badge X2 (1.4 kg)
Sooty Blotch/Flyspeck	PF + Covers	16 May, 6 and 23 June	-	-	Cueva (4.67 L) + Double Nickel (2.33 L)	Microthiol Disperss (16.8 kg)
	Covers	17 July, 6 and 22 August, 15 September	28 May, 6 and 25 June, 17 July, 5 August	3 and 24 June, 26 July, 15 August	Cueva (4.67 L) + Double Nickel (2.33 L)	Microthiol Disperss (16.8 kg)

OBLR, obliquebanded leafroller; PLH, potato leafhopper; PF, petal fall. ^a^ Ecover detergent (1.17 L) added to Neem oil sprays.

**Table 2:** Obliquebanded leafroller and fruit-feeding Lepidoptera in-season damage.

Year/Treatment ^1^	Percent Trees with OBLR Larval Infestations		Percent Damaged Fruits—Int Leps
2014	22 May	4 June	23 July
Advanced Organic	4.0 ± 0.4	0.7 ± 0.3	1.0 ± 0.4 a
Minimal Organic	3.6 ± 0.7	1.2 ± 0.3	2.4 ± 0.5 a,b
Untreated Control	4.7 ± 0.8	1.2 ± 0.3	4.4 ± 1.5 b
2015	9 June		16 July
Advanced Organic	0.2 ± 0.1		1.2 ± 0.3 a
Minimal Organic	0.6 ± 0.2		2.3 ± 0.6 a,b
Untreated Control	0.7 ± 0.3		3.5 ± 0.8 b
2016	2 June		
Advanced Organic	3.8 ± 1.1 a		
Minimal Organic	8.7 ± 2.8 a,b		
Untreated Control	13.7 ± 3.1 b		

OBLR, obliquebanded leafroller; Int Leps, internal-feeding Lepidoptera. ^1^ Within a year, values within a pest category followed by the same letter are not significantly different, *p* < 0.05, Student’s *t*-test.

**Table 3:** Green aphid and potato leafhopper terminal infestations under different organic programs.

Year/Treatment ^1^	Percent Aphid Infestations				
2014	17 June	24 June	2 July	16 July	23 July
Advanced Organic	12.6 ± 3.2	6.7 ± 1.7	9.3 ± 2.9	2.6 ± 1.2	5.9 ± 1.5
Minimal Organic	8.5 ± 2.5	3.3 ± 1.2	8.5 ± 2.1	2.6 ± 1.1	3.3 ± 1.2
Untreated Control	7.9 ± 2.0	7.0 ± 1.6	4.5 ± 1.8	8.1 ± 1.9	7.8 ± 1.4
	Percent PLH inf	Percent aphid infestations			
2015	30 June	30 June	16 July	22 July	11 August
Advanced Organic	25.6 ± 3.2	0.7 ± 0.5	1.9 ± 1.0	2.6 ± 1.2	5.9 ± 1.6
Minimal Organic	21.1 ± 2.5	1.1 ± 0.8	4.8 ± 1.8	3.7 ± 1.2	7.8 ± 2.4
Untreated Control	22.6 ± 2.0	1.1 ± 0.7	3.3 ± 1.2	2.1 ± 0.7	7.3 ± 2.3
	Percent PLH infestations		Percent aphid infestations		
2016	21 June	1 July	17 June	21 June	1 July
Advanced Organic	33.0 ± 10.6	0.0 a	12.6 ± 3.2	5.6 ± 1.8	1.1 ± 1.1
Minimal Organic	36.7 ± 7.3	0.0 a	8.5 ± 2.5	2.7 ± 1.1	2.2 ± 2.2
Untreated Control	38.7 ± 5.7	24.4 ± 12.5 b	9.6 ± 2.0	3.3 ± 0.8	0.0

PLH inf, potato leafhopper infestations. ^1^ Within a year, infestation levels followed by the same letter not significantly different, *p* < 0.05, Student’s *t*-test.

**Table 4:** Percent fruit insect damage at harvest under different organic programs, 2014–2016.

Year/Treatment ^1^	Plum Curculio Oviposition	Plum Curculio Feeding	Internal Lepidoptera	Tarnished Plant Bug	Rosy Apple Aphid	Early OBLR	Late OBLR	San Jose Scale	Stink Bug	European Apple Sawfly	Clean Fruit
2014											
Advanced Organic	13.8 ± 3.7	10.0 ± 1.5	0.8 ± 0.3	3.2 ± 0.9	1.7 ± 0.8	**-**	9.2 ± 2.9 a	**-**	**-**	**-**	62.6 ± 3.2
Minimal Organic	12.5 ± 4.3	8.0 ± 0.8	0.9 ± 0.4	2.5 ± 0.7	1.3 ± 0.7	**-**	16.1 ± 1.8 a,b	**-**	**-**	**-**	59.7 ± 4.0
Untreated Control	13.6 ± 3.7	7.6 ± 2.1	4.0 ± 2.1	3.1 ± 0.8	1.7 ± 1.0	**-**	22.8 ± 5.3 b	**-**	**-**	**-**	51.5 ± 5.5
2015											
Advanced Organic	8.4 ± 2.3 a	3.9 ± 2.1	8.4 ± 1.1 a	9.6 ± 1.7	4.2 ± 2.0 a	0.4 ± 0.2 a	4.8 ± 1.1	2.6 ± 1.5	**-**	**-**	60.8 ± 2.6 a
Minimal Organic	20.8 ± 5.8 a,b	3.7 ± 2.6	19.8 ± 2.1 b	9.0 ± 2.5	0.7 ± 0.3 b	0.6 ± 0.4 a	6.6 ± 1.4	0.6 ± 0.4	**-**	**-**	44.3 ± 4.2 b
Untreated Control	26.9 ± 5.6 b	2.8 ± 1.7	13.9 ± 32.3 a,b	9.3 ± 1.9	2.4 ± 0.9 a,b	2.8 ± 0.5 b	8.1 ± 1.7	2.3 ± 0.8	**-**	**-**	42.4 ± 5.8 b
2016											
Advanced Organic	14.2 ± 5.4 a	12.9 ± 3.4 a	8.7 ± 1.3	3.4 ± 1.0	0.0	0.0	4.8 ± 0.7	7.5 ± 3.5 a	2.5 ± 1.9	0.0	50.5 ± 6.8 a
Minimal Organic	28.5 ± 9.6 a,b	20.0 ± 3.0 a,b	16.0 ± 4.8	2.2 ± 0.7	0.9 ± 0.7	0.0	5.2 ± 0.4	0.4 ± 0.4 b	3.7 ± 3.5	0.2 ± 0.2	33.1 ± 8.9 a,b
Untreated Control	39.8 ± 6.9 b	31.0 ± 9.1 b	15.7 ± 3.4	4.9 ± 3.1	2.0 ± 2.0	0.4 ± 0.3	8.2 ± 2.7	1.8 ± 1.0a b	6.4 ± 6.4	0.6 ± 0.6	16.6 ± 5.3 b

Plum curculio, *Conotrachelus nenuphar* (Herbst); internal Lepidoptera: complex including *Cydia pomonella* (L.), *Grapholita molesta* (Busck) or *G. prunivora* (Walsh); tarnished plant bug, *Lygus lineolaris* (Palisot de Beauvois); rosy apple aphid, *Dysaphis plantaginea* (Passerini); OBLR, obliquebanded leafroller, *Choristoneura rosaceana* (Harris); San Jose scale, *Quadraspidiotus perniciosus* (Comstock); stink bug, spp. undetermined but likely either *Acrosternum hilare* (Say) or *Euschistus servus* (Say); European apple sawfly, *Hoplocampa testudinea* (Klug).^1^ Within a year, percent fruit levels within a pest category followed by the same letter not significantly different, *p* < 0.05, Student’s *t*-test.

**Table 5:** Development of sooty blotch and flyspeck in selected varieties under different organic management programs, 2014–2016.

Treatment/year	CC1009 ^4^	Crimson Crisp	Gold Rush	Juliet	Modi	Topaz
2014 ^1^						
Advanced Organic	42.7 ± 4.7 b	19.7 ± 2.7 b	2.0 ± 1.2 c	8.0 ± 0.0 b	7.5 ± 2.6 b	8.7 ± 4.7 b
Minimal Organic	58.7 ± 7.5 a,b	11.3 ± 2.4 b	27.6 ± 4.6 b	7.3 ± 0.0 b	8.8 ± 1.9 b	6.7 ± 4.1 b
Untreated Control	64.0 ± 9.2 a	44.0 ± 4.2 a	76.7 ± 7.7 a	59.3 ± 0.0 a	46.7 ± 5.5 a	81.3 ± 6.6 a
2015 ^2^						
Advanced Organic	32.0 ± 5.8 b	4.0 ± 2.3 b	7.3 ± 3.5 b	15.3 ± 2.9 b	14.0 ± 3.1 b	44.0 ± 4.0 b
Minimal Organic	32.0 ± 7.2 b	9.3 ± 2.7 b	6.7 ± 1.8 b	8.7 ± 2.9 b	8.7 ± 5.9 b	14.7 ± 1.8 c
Untreated Control	84.7 ± 8.4 a	62.7 ± 9.7 a	100.0 ± 0.0 a	80.0 ± 4.6 a	83.3 ± 1.8 a	100.0 ± 0.0 a
2016 ^3^						
Advanced Organic	10.0 ± 4.0 b	4.7 ± 1.8 b	2.7 ± 0.7 c	2.7 ± 1.3 b	0.0 b	5.3 ± 0.7 b
Minimal Organic	14.0 ± 0.0 b	2.0 ± 1.2 b	20.7 ± 0.7 b	5.3 ± 0.7 b	8.7 ± 0.7 b	16.0 ± 1.1 b
Untreated Control	36.7 ± 4.1 a	25.3 ± 1.3 a	43.3 ± 5.9 a	27.3 ± 5.2 a	30.7 ± 0.7 a	48.0 ± 3.5 a

^1^ Treatment programs in 2014 (amt/ha): Advanced, Cueva (7.0 L) + Double Nickel LC (2.33 L). Minimal, Microthiol Disperss (16.8 kg). Application timings: 17 July, 6 August, 22 August, 15 September. ^2^ Treatment programs in 2015 (amt/ha): Advanced, Badge X2 (5.6 kg) 22 April; Badge X2 (1.4 kg) 8, 12, and 15 May; Microthiol Disperss (16.8 kg); application timings: 28 May, 6 and 25 June, 17 July, 5 August. Minimal, Cueva (7.0 L) 22 April, 8 May; Cueva (4.67 L) + Double Nickel LC (2.33 L); application timings: 12, 15 and 28 May, 6 and 25 June, 17 July, 5 August. ^3^ Treatment programs in 2016 (amt/A): Advanced, Badge X2 (5.6 kg) 19 April; Badge X2 (1.4 kg) 6, 11, and 16 May; Microthiol Disperss (16.8 kg); application timings: 24 May, 9 June, 8 July, 9 August. Minimal, Cueva (7.0 L) 19 April, 6 May; Cueva (4.67 L) + Double Nickel LC (2.33 L); application timings: 11, 16, and 24 May, 9 June, 8 July, 9 August. ^4^ Incidence of sooty block and sooty blotch on mature fruit at harvest. All values represent the means and standard errors of five fruit with ten collections from each of 15 trees in three replicate plots. Values within columns followed by the same letter are not significantly different (*p* < 0.05) according to the LSMEANS procedure in SAS 9.4 with an adjustment for Tukey’s HSD to control for family-wise error.

**Table 6:** Development of cedar apple rust on terminal leaves in selected varieties under different organic management programs, 2015–2016.

Treatment/year	CC1009 ^3^	Crimson Crisp	Goldrush	Juliet	Modi	Topaz	Pristine
2015 ^1^							
Advanced Organic	0.0	19.3 ± 0.7 b	20.7 ± 3.7 b	13.3 ± 5.3 b	0.0	18.0 ± 2.0 b	6.7 ± 2.9 b
Minimal Organic	0.0	17.3 ± 11.6 b	22.7 ± 4.8 b	8.7 ± 2.7 b	0.0	15.3 ± 5.5 b	0.7 ± 0.7 b
Untreated Control	0.0	77.3 ± 3.3 a	72.7 ± 3.3 a	38.0 ± 3.1 a	0.0	64.0 ± 1.2 a	26.7 ± 4.8 a
2016 ^2^							
Advanced Organic	0.0	7.3 ± 1.3 b	10.0 ± 5.3 b	2.0 ± 0.0 b	0.0	4.7 ± 0.6 b	0.0 b
Minimal Organic	0.0	11.3 ± 3.3 b	10.0 ± 2.0 b	6.0 ± 2.0 b	0.0	10.7 ± 1.8 b	1.3 ± 0.7 b
Untreated Control	0.0	34.7 ± 7.5 a	32.0 ± 4.0 a	22.7 ± 2.4 a	0.0	24.7 ± 4.1 a	10.7 ± 0.7 a

^1^ Treatment programs in 2015 (amt/ha): Advanced, Badge X2 (5.6 kg) 22 April; Badge X2 (1.4 kg) 8, 12, and 15 May; Microthiol Disperss (16.8 kg); application timings: 28 May, 6 and 25 June, 17 July, 5 August. Minimal, Cueva (7.0 L) 22 April, 8 May; Cueva (4.67 L) + Double Nickel LC (2.33 L); application timings: 12, 15 and 28 May, 6 and 25 June, 17 July, 5 August. ^2^ Treatment programs in 2016 (amt/ha): Advanced, Badge X2 (5.6 kg) 19 April; Badge X2 (1.4 kg) 6, 11, and 16 May; Microthiol Disperss (16.8 kg); application timings: 24 May, 9 June, 8 July, 9 August. Minimal, Cueva (7.0 L) 19 April, 6 May; Cueva (4.67 L) + Double Nickel LC (2.33 L); application timings: 11, 16, and 24 May, 9 June, 8 July, 9 August. ^3^ Incidence of cedar apply rust lesions on terminal leaves. All values represent the means and standard errors of eight terminal leaves from ten shoot from each of 15 trees from three replicate plots. Values within columns followed by the same letter are not significantly different (*p* < 0.05) according to the LSMEANS procedure in SAS 9.4 with an adjustment for Tukey’s HSD to control for family-wise error.

**Table 7:** Development of blossom blight in selected varieties under different organic management programs, 2015–2016.

Treatment/year	Goldrush ^3^	Modi	Topaz
**2015 ^1^**			
Advanced Organic	0.0 ± 0.0 b	1.0 ± 0.7 b	1.8 ± 0.5 b
Minimal Organic	1.0 ± 0.4 b	0.0 ± 0.0 b	0.5 ± 0.5 b
Untreated Control	7.3 ± 0.5 a	3.4 ± 0.5 a	5.6 ± 1.0 a
**2016 ^2^**			
Advanced Organic	1.3 ± 0.5 b	0.8 ± 0.3 b	0.5 ± 0.3 b
Minimal Organic	7.8 ± 1.4 b	2.8 ± 0.5 b	5.0 ± 0.4 b
Untreated Control	23.3 ± 1.9 a	14.0 ± 1.7 a	21.8 ± 2.8 a

^1^ Treatment programs in 2015 (amt/A): Advanced, Badge X2 (5.6 kg) 22 April; Badge X2 (1.4 kg) 8, 12, and 15 May; Microthiol Disperss (16.8 kg); application timings: 28 May, 6 and 25 June, 17 July, 5 August. Minimal, Cueva (7.0 L) 22 April, 8 May; Cueva (4.67 L) + Double Nickel LC (2.33 L); application timings: 12, 15 and 28 May, 6 and 25 June, 17 July, 5 August. ^2^ Treatment programs in 2016 (amt/A): Advanced, Badge X2 (5.6 kg) 19 April; Badge X2 (1.4 kg) 6, 11, and 16 May; Microthiol Disperss (16.8 kg); application timings: 24 May, 9 June, 8 July, 9 August. Minimal, Cueva (7.0 L) 19 April, 6 May; Cueva (4.67 L) + Double Nickel LC (2.33 L); application timings: 11, 16, and 24 May, 9 June, 8 July, and 9 August. ^3^ Incidence of blossom bight two weeks after inoculation. All values represent the means and standard errors of five flower 5 clusters with 20 cluster assessments from each of 15 trees from three replicate plots. Values within columns followed by the same letter are not significantly different (*p* < 0.05) according to the LSMEANS procedure in SAS 9.4 with an adjustment for Tukey’s HSD to control for family-wise error.

**Table 8:** Costs of arthropod and disease control products sprayed under different organic management programs, 2014–2016.

Treatment/year	Cost per ha, US$		
	Arthropods	Diseases	Total
2014			
Advanced Organic	$1166	$558	$1724
Minimal Organic	$1088	$375	$1463
2015 and 2016			
Advanced Organic	$1344	$1032	$2376
Minimal Organic	$1176	$514	$1690
